# Nutritional Approaches Targeting Gut Microbiota in Oxidative-Stress-Associated Metabolic Syndrome: Focus on Early Life Programming

**DOI:** 10.3390/nu16050683

**Published:** 2024-02-28

**Authors:** You-Lin Tain, Chien-Ning Hsu

**Affiliations:** 1 Division of Pediatric Nephrology, Kaohsiung Chang Gung Memorial Hospital, Kaohsiung 833, Taiwan; tainyl@cgmh.org.tw; 2 Institute for Translational Research in Biomedicine, Kaohsiung Chang Gung Memorial Hospital, Kaohsiung 833, Taiwan; 3 College of Medicine, Chang Gung University, Taoyuan 333, Taiwan; 4 Department of Pharmacy, Kaohsiung Chang Gung Memorial Hospital, Kaohsiung 833, Taiwan; 5 School of Pharmacy, Kaohsiung Medical University, Kaohsiung 807, Taiwan

**Keywords:** hypertension, metabolic syndrome, developmental origins of health and disease (DOHaD), oxidative stress, gut microbiota, short-chain fatty acid, probiotics, nitric oxide

## Abstract

Metabolic syndrome (MetS) denotes a constellation of risk factors associated with the development of cardiovascular disease, with its roots potentially traced back to early life. Given the pivotal role of oxidative stress and dysbiotic gut microbiota in MetS pathogenesis, comprehending their influence on MetS programming is crucial. Targeting these mechanisms during the early stages of life presents a promising avenue for preventing MetS later in life. This article begins by examining detrimental insults during early life that impact fetal programming, ultimately contributing to MetS in adulthood. Following that, we explore the role of oxidative stress and the dysregulation of gut microbiota in the initiation of MetS programming. The review also consolidates existing evidence on how gut-microbiota-targeted interventions can thwart oxidative-stress-associated MetS programming, encompassing approaches such as probiotics, prebiotics, postbiotics, and the modulation of bacterial metabolites. While animal studies demonstrate the favorable effects of gut-microbiota-targeted therapy in mitigating MetS programming, further clinical investigations are imperative to enhance our understanding of manipulating gut microbiota and oxidative stress for the prevention of MetS.

## 1. Introduction

Metabolic syndrome (MetS), characterized by a combination of conditions such as obesity, insulin resistance, hypertension, and dyslipidemia, stands as a paramount threat to global health. It not only contributes to two-thirds of non-communicable disease (NCD) deaths worldwide, but also escalates the risk of cardiovascular disease (CVD) [1]. Despite varied definitions of MetS [2], its global prevalence is estimated to impact approximately one-quarter of the global population [1]. While several treatments have been applied clinically for diverse MetS phenotypes, a notable absence of specific therapeutic regimens persists, emphasizing the need for preventive interventions to curb the rising prevalence of MetS alongside treatment-focused approaches [3].

Emerging evidence suggests that the early-life environment can exert enduring effects on lifelong human well-being. Vulnerability to MetS may initiate with events occurring in the prenatal and infancy phases, leaving lasting impacts in relation to MetS and its associated complications in adulthood [4,5,6]. Referred to as the developmental origin of health and disease (DOHaD) [7], this theory underscores how early-life environmental exposures shape later health and disease risk. Consequently, efforts have been directed towards identifying early-life risk factors, preventing their exposures, particularly during pregnancy and lactation, and exploring interventions to enhance long-term outcomes, known as reprogramming.

Several early-life risk factors, often associated with adverse in utero environments, may contribute to susceptibility to adult MetS [8,9]. However, accumulating evidence suggests that the interplay between oxidative stress and dysbiosis in the gut microbiota is pivotal in the developmental programming of MetS.

Compelling evidence indicates that the mechanisms underlying MetS risk factors may originate in the gut microbiome [10]. Interactions within the gut microbiota community can process external cues contributing to major MetS components, such as obesity, hyperglycemia, dyslipidemia, and hypertension [10]. Furthermore, recent findings implicate gut-microbial-derived metabolites like tryptophan-derived uremic toxins and trimethylamine N-oxide (TMAO) in elevating CVD risk [11,12]. The intricate connection between diet, gut microbiota, and metabolic outcomes is well established [13], linking maternal imbalanced diets to developmental origins of MetS through disruptions in gut microbiota composition or function.

Oxidative stress arises when an excess of reactive oxygen species (ROS) overwhelms the antioxidant defense system, and its involvement in the etiology of various disorders has been uncovered [14]. The involvement of oxidative stress in MetS is evident as both a contributing factor and a resultant condition. Although the acknowledged role of oxidative stress in the pathogenesis of MetS is widespread [15], additional clarification is required to fully understand its causative effects on the programming of MetS.

**Figure 1:**
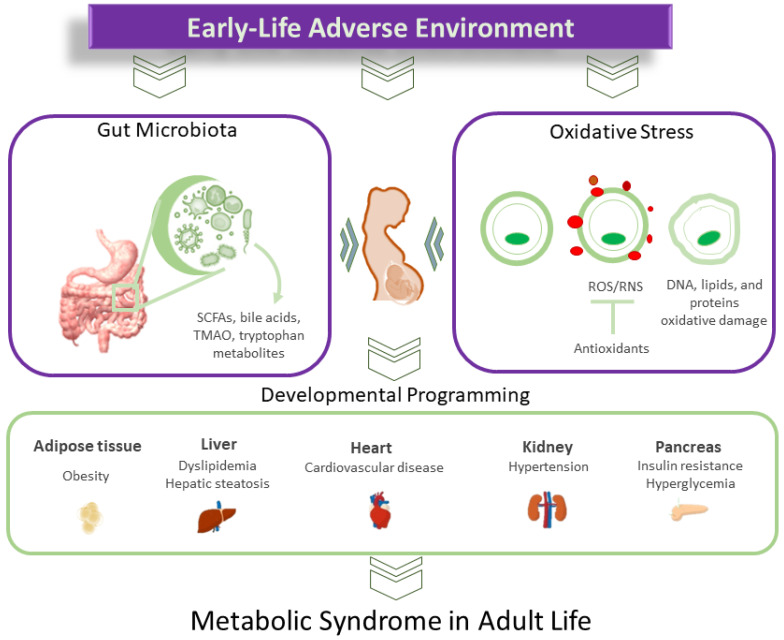
Diagram illustrating the intricate connections between oxidative stress and gut microbiota in the developmental programming of metabolic syndrome.

A balanced gut microbiome depends on a stable redox balance, and gut microbiota dysbiosis disrupts this equilibrium. There is growing interest in targeting the gut microbiota through diet and nutritional approaches. This review aims to delve deeper into the interplay between oxidative stress and gut microbiota and their impact on the developmental origins of MetS. We discuss potential mechanisms involved in MetS development, focusing on the role of oxidative stress and gut microbiota dysbiosis. Subsequently, we summarize nutritional approaches targeting gut microbiota in animal models to prevent oxidative-stress-associated MetS programming. Gut-microbiota-targeted therapies have been employed clinically to address obesity and associated disorders. However, there is a notable lack of information regarding their potential application in the context of MetS programming in humans within the field of DOHaD. Figure 1 illustrates the interrelationships between early-life adverse environments, oxidative stress, gut microbiota, and the developmental programming of MetS.

## 2. MetS of Developmental Origins

Several lines of epidemiological evidence have substantiated the link between adverse early-life conditions and the heightened risk of offspring developing MetS. First, research indicates that offspring exposed to famines exhibit an increased susceptibility to developing MetS [16,17,18]. The Dutch Famine Birth Cohort Study demonstrated that maternal famine exposure programmed offspring towards conditions such as obesity, dyslipidemia, hypertension, insulin resistance, and CVD in adulthood [18]. Another strand of evidence stems from twin pregnancy studies, revealing an association between low birth weight (LBW) and specific MetS characteristics as twins mature [19,20]. Additionally, numerous studies have established that LBW correlates with various aspects of adult MetS, including hypertension [21], obesity [22], insulin resistance [23], non-alcoholic fatty liver disease (NAFLD), and dyslipidemia [24]. Notably, given that CVD is a significant complication of MetS, there is a notable surge in CVD risk associated with LBW [25]. Lastly, various early-life risk factors linked to subsequent MetS have been identified in observational studies. These factors during pregnancy encompass environmental chemical exposure [26], maternal obesity [27], gestational diabetes [28], cigarette smoke [29], and maternal stress [30]. In addition to maternal factors, adverse cardiometabolic outcomes in offspring are associated with paternal obesity, advanced paternal age at conception, paternal diabetes mellitus, and paternal smoking [31,32].

While human studies establish a connection between adverse in utero environments and later-life MetS, the underlying molecular mechanisms of MetS programming necessitate investigation to devise successful reprogramming strategies. The exploration of innovative interventions has been significantly aided by the pivotal role played by animal models in establishing the biological plausibility of these concepts [6,9].

To emulate facets of MetS observed in humans, various adverse early-life environments, such as maternal nutritional deficiencies and imbalances [9,33], maternal medical issues [34,35], exposure to environmental chemicals [36,37], and medication use [38,39], have been examined to create animal models of offspring MetS [6]. It is noteworthy that, currently, no single animal model fully replicates all features of human MetS. Most research on MetS programming employs models displaying specific components of MetS [6]. Although a broad range of maternal insults are linked to certain MetS features, only some animal models present two or more components of MetS in adult offspring [6]. In addition to commonly used rats, other animals, including mice [40], sheep [41], swine [42], rabbits [43], and non-human primates [44], have been utilized as animal models for studying MetS programming.

To date, animal studies have shed light on several mechanistic frameworks proposed to elucidate the biological basis of MetS programming, including oxidative stress [45,46], dysbiotic gut microbiota [47], impaired nutrient sensing [48], epigenetic regulation [49], aberrant activation of the renin–angiotensin system (RAS) [50], deficient nitric oxide (NO) [51], and glucocorticoid programming [52].

While the core mechanisms involved in MetS programming still require further exploration, early-life adverse insults may mediate these interconnected mechanisms and impact multiple interacting organ systems, ultimately leading to adult MetS. The primary emphasis of this review centers on rat models concerning MetS programming associated with oxidative stress and gut microbiota dysbiosis. A more in-depth discussion of these models will follow in the subsequent section.

## 3. Oxidative Stress and MetS of Developmental Origins

### 3.1. Oxidative Stress during Pregnancy

Throughout pregnancy, maintaining a delicate balance between reactive oxygen species (ROS) and antioxidants is crucial to create an optimal environment for the developing fetus [53]. ROS, while playing a pivotal role in the regulation of transcription factors and signaling pathways essential for fetal growth and development, can become a double-edged sword. Normal ROS levels are necessary for these processes, but an excess of ROS can lead to oxidative stress, causing cellular damage. Factors contributing to increased ROS production include elevated oxygen consumption, heightened metabolism, and an increased utilization of fatty acids. The excessive production of ROS disrupts these vital processes, leading to complications in maternal health during pregnancy [54].

Oxidative stress plays a significant role in the inactivation of nitric oxide (NO) as a result of excessive ROS. NO, a well-known vasodilator, governs much of the feto-placental circulation [55]. The production of NO can be inhibited by its endogenous inhibitor, asymmetric dimethylarginine (ADMA). ADMA levels decrease in the first trimester and then rise as gestational age increases [56,57]. This physiological reduction in ADMA, accompanied by high NO levels, represents a hemodynamic adaptation in early pregnancy to meet the heightened need for organ perfusion and maintain uterine relaxation. Conversely, an increase in ADMA in later gestation supports higher uterine muscle contractile activity, which is crucial for successful delivery [58].

Several maternal medical conditions and complications of pregnancy, now recognized to induce oxidative stress, encompass maternal undernutrition, obesity, preeclampsia, gestational diabetes, intrauterine growth retardation (IUGR), and maternal smoking [45,59,60,61]. Notably, gestational diabetes, preeclampsia, and maternal undernutrition are associated with elevated ADMA levels. In summary, these observations highlight that an imbalance between ROS and NO leads to oxidative stress, a fundamental mechanism participating in fetal programming during compromised pregnancies.

### 3.2. Oxidative Stress in Animal Models of MetS of Developmental Origins

While the notion of oxidative stress playing a pathogenic role in MetS has been suggested [15], there is a limited number of studies delving into the consequences of early-life oxidative stress on the emergence of MetS in offspring. Table 1 offers a comprehensive summary of the current literature, spotlighting investigations that examine oxidative stress in animal models displaying at least two features of MetS in their offspring [61,62,63,64,65,66,67,68,69,70,71,72,73,74,75,76,77,78,79,80,81,82,83,84,85,86,87,88,89,90,91,92,93,94,95,96,97,98,99,100,101,102,103,104,105,106,107]. Given their relatively shorter life span, these investigations have predominantly utilized rat models. The table includes the age at which offspring were assessed, with the age range for rats spanning from 12 to 52 weeks, equivalent to young to middle adulthood in humans [108].

Various adverse maternal conditions have been utilized to induce rodent models of MetS programming. These conditions encompass maternal undernutrition, maternal overnutrition, maternal illness, pregnancy complications, maternal smoking, and chemical exposure. Protocols involving caloric or protein restriction have been employed to simulate human famine research. Maternal caloric restriction has been shown to result in hypertension and insulin resistance in adult progeny [61,62,63]. Similarly, offspring from dams exposed to protein restriction exhibited elevated blood pressure (BP) and insulin resistance [64,65]. Conversely, MetS programming can also be initiated by maternal overnutrition. Offspring exposed to maternal high fructose intake demonstrated hypertension, insulin resistance, dyslipidemia, and obesity [66,67,68]. Similarly, a maternal diet rich in saturated fat led to programmed hypertension, obesity, dyslipidemia, and hyperinsulinemia [72,73,74,75,76,77].

Maternal diseases and complications during pregnancy have been linked to offspring MetS. For instance, the offspring of diabetic rat dams exhibited hypertension, obesity, insulin resistance, and dyslipidemia [78,79,80]. Although various animal models of maternal diabetes have been developed, only streptozotocin (STZ)-induced diabetes has been explored in the context of MetS of developmental origins [78,79,80]. Maternal inflammation induced by lipopolysaccharide (LPS) and maternal uteroplacental insufficiency have been associated with hypertension and insulin resistance in adult progeny [83,84,85,86,87]. Additionally, maternal stress induced by excessive glucocorticoids has been studied in relation to offspring MetS, revealing associations with hypertension, obesity, and insulin resistance following maternal dexamethasone exposure [89,90,91].

Exposure to environmental toxins/chemicals during pregnancy has also been implicated in the developmental programming of MetS. Perinatal nicotine exposure has been linked to hypertension, hyperlipidemia, and steatosis in adult offspring [97,98,99]. Pregnant rats intubated with 1 g of ethanol/kg at days 13 and 14 of gestation exhibited hypertension and insulin resistance in offspring at 6 months of age [100,101]. Furthermore, maternal exposure to bisphenol A (BPA) [103,104] or di-n-butyl phthalate (DEHP) [105,106] resulted in an increase in BP and insulin resistance in adult rat offspring.

### 3.3. Mechanisms of Oxidative Stress

Mechanisms underlying oxidative stress encompass a range of processes that culminate in an overproduction of reactive oxygen species (ROS), potentially causing harm to cellular components. Key mechanisms include mitochondrial dysfunction, inflammation, metal ion imbalance, oxidative enzymatic reactions (such as NADPH oxidase and xanthine oxidase), ischemia–reperfusion injury, endoplasmic reticulum stress, lipid peroxidation, DNA damage, and compromised antioxidant defenses [14,15,53]. These factors collectively contribute to the intricate web of events associated with oxidative stress, impacting cellular health and function. The mechanisms underlying increased oxidative stress in MetS programming are not fully elucidated, but they involve the heightened expression of NADPH oxidase [92,97,102], elevated levels of ROS [71,82,88,96,107], diminished activity of antioxidant enzymes [64,76,82,92,99,102], increased peroxynitrite [62,81], elevated ADMA [61,93,103], and reduced NO [61,62,69,78,89,103]. In MetS of developmental origins, oxidative stress can manifest in various organs, including the brain [70,96], kidneys [61,85], vessels [62], and liver [99].

The impaired removal of ROS due to reduced antioxidant capacities is a contributing factor to oxidative-stress-associated MetS programming. Models of MetS programming have demonstrated a decrease in glutathione [64] and a reduction in the activities of antioxidant enzymes such as SOD [76,82,102], glutathione peroxidase 1 [92,99], and catalase [102]. Numerous specific markers of lipid, protein, and DNA oxidative damage have been investigated in various programmed MetS models, including malondialdehyde (MDA) [76,85,97], F2-isoprostanes [64,88], thiobarbituric acid reactive substances (TBARS) [81], 4-hydroxynonenal (4-NHE) [99], and 8-hydroxydeoxyguanosine (8-OHdG) [61,69,77,103].

The disruption of the ADMA/NO pathway is another oxidative-stress-associated mechanism involved in MetS programming. Table 1 highlights the crucial role of ADMA in several models of oxidative-stress-associated MetS programming, such as caloric restriction [61], maternal diabetes [78], maternal stress [93], and BPA exposure [103]. Likewise, deficient NO levels were observed in these models.

## 4. Gut Microbiota and MetS of Developmental Origins

### 4.1. Gut Microbiota

The gut microbiota constitutes a symbiotic community comprising trillions of microbes belonging to over 1000 species actively involved in modulating the host’s physiological functions. These functions include regulating gut homeostasis, influencing the absorption and metabolism of dietary nutrients, impacting the immune system, controlling BP, and contributing to drug metabolism [109]. The composition of the gut microbiota is highly individualized and undergoes transformations throughout the human lifespan, influenced by factors such as diet, lifestyle, medications, environment, and various diseases [109]. Despite the presence of some evidence that supports the transmission of microbes from mothers to fetuses during pregnancy, the existence of the prenatal microbiome has been a topic of considerable debate in recent years [110]. Microbial colonization of the neonatal gut begins shortly after birth, with the infant microbiota achieving an adult-like composition by the age of 2–3 years [111]. The mother’s microbiome significantly influences the composition of the offspring’s gut microbiome [112]. Notably, multiple factors, including maternal conditions, feeding type, delivery method, antibiotic exposure, ecological factors, and gestational age, are associated with the composition of the offspring’s gut microbiome [112].

Several of the aforementioned factors implicated in the developmental origins of MetS have been linked to disturbances in the gut microbiota. The interactions between the host and microbes are mediated by microbial-derived metabolites [113,114]. Dietary-derived bacterial metabolites, particularly TMAO, short-chain fatty acids (SCFAs), tryptophan derivatives, bile acids, and branched-chain amino acids, play a significant role in maintaining metabolic homeostasis.

### 4.2. Gut Microbiota Dysbiosis and MetS Programming

An imbalance in the composition of gut microbiota, known as dysbiosis, contributes to the fundamental pathogenesis of MetS programming [10]. Numerous components of offspring MetS, such as hypertension [115], obesity [116], insulin resistance [117], and dyslipidemia [118], have been documented in association with dysbiosis.

A maternal high-fat diet is often employed to investigate MetS programming, as this model induces various MetS components in adult offspring [6]. The long-term consequences of a maternal high-fat diet include a reduction in α-diversity in the offspring’s microbiota [119]. Dysbiosis characterized by a loss of α-diversity is associated with several human diseases [120]. Additionally, a maternal high-fat diet induces offspring hypertension, linked to an elevated *Firmicutes*-to-*Bacteroidetes* (F/B) ratio. An increased F/B ratio, resulting from *Firmicutes* expansion and/or *Bacteroidetes* contraction, is widely considered a hallmark of cardiometabolic disease [121]. Gut dysbiosis may also stem from a depletion of beneficial microbes [109], with previous studies indicating a reduced abundance of beneficial bacterial strains like *Lactobacillus* and *Akkermansia* in adult offspring born to dams fed a high-fat diet [122,123]. It has been shown that a maternal high-fat diet induces offspring hypertension, accompanied by alterations in gut microbiota content, reduced fecal SCFAs, dysregulated SCFA receptors, and impaired TMAO metabolic pathways in adult rat offspring [122].

Gut dysbiosis increases intestinal permeability, leading to lipopolysaccharide (LPS) absorption, endotoxemia, and systemic inflammation [124]. Elevated circulating LPS, generated in the intestine, is implicated in the pathogenesis of insulin resistance and obesity [125]. Prenatal exposure to LPS has been shown to lead to offspring hypertension [83]. Feeding lactating dams with low-fiber diets results in offspring with microbiota dysbiosis, characterized by an impaired gut barrier, increased bacterial translocation, reduced taxonomic diversity, and an increased proportion of *Proteobacteria* species. This microbiota dysbiosis is associated with low-grade gut inflammation and increased adiposity in adult offspring [126].

Gut microbiota dysbiosis also affects bile acid metabolism. Bile acids act as signaling molecules that regulate lipid digestion, cholesterol metabolism, and other regulatory pathways. Dysregulated gut-microbiota-derived bile acids and their receptors are implicated in type 2 diabetes, obesity, dyslipidemia, and NAFLD [127]. As an example, a diet rich in fats has been shown to induce hyperlipidemia, linked to compromised bile acid metabolism [128]. In another investigation, it was observed that maternal high-fat consumption led to NAFLD in offspring, accompanied by alterations in bile acid composition [129].

Several tryptophan derivatives produced by gut microbes may contribute to MetS pathogenesis [130]. The gut microbiota controls the three major tryptophan metabolic pathways leading to kynurenine, indole, and serotonin derivatives. Increased kynurenine levels are correlated with obesity and hyperlipidemia [131], while decreased serotonin levels are negatively correlated with BMI and body fat in patients with MetS [132]. Additionally, indole catabolites of tryptophan in the microbial metabolism participate in MetS pathogenesis via activating the aryl hydrocarbon receptor (AHR) signaling pathway [130,133]. Impaired AHR signaling is associated with various MetS components, including obesity [134], insulin resistance [135], liver steatosis [136], and hypertension [137]. Moreover, gut microbiota regulation of the bile acid metabolism is crucial for maintaining a healthy gut microbiota, insulin sensitivity, and balanced carbohydrate/lipid metabolism [138]. Studies in both humans and animals have shown that MetS is associated with the dysregulation of bile acid homeostasis [139]. Conversely, microbiota dysbiosis may be modulated through the administration of dietary nutrients, prebiotics, or probiotics to help treat MetS [140].

## 5. Targeting Gut Microbiota to Reprogram MetS

Implementing early-life interventions to reprogram adult disease represents a promising strategy for disrupting adverse programming processes [141]. Given the significant role of gut microbiota in the developmental origins of MetS, targeted therapies focusing on the gut microbiota could serve as a reprogramming approach to mitigate the risk of MetS in adulthood.

### 5.1. Gut-Microbiota-Targeted Therapy

Considering the intricate relationship between gut microbiota and host health, concerted efforts have been directed towards implementing diverse therapies aimed at modulating the gut microbiota to manage or prevent various diseases, including MetS [142,143,144].

MetS is notably influenced by insufficient dietary habits, as demonstrated by the Western diet. Conversely, the preservation of gut microbiota and protection against MetS are closely associated with the maintenance of a well-balanced and healthy diet [145]. Diets rich in high-fiber foods, plant-based ingredients, and fermented foods are linked to a more diverse and advantageous gut microbiota, ultimately contributing to improved cardiometabolic health [145].

An illustrative example is the Mediterranean diet, which draws inspiration from the traditional eating habits of individuals in the Mediterranean region [146]. This dietary approach, while limiting the intake of processed foods, underscores the importance of a high consumption of vegetables, fruits, whole grains, seafood, and olive oil [147]. The consistent incorporation of these elements into the Mediterranean diet results in the accumulation of polyunsaturated fatty acids (PUFAs) and polyphenolic compounds in the human body. Notably, one captivating aspect of the Mediterranean diet is its positive influence on gut microbiota.

Moreover, the Dietary Approaches to Stop Hypertension (DASH) diet, characterized by a high intake of fiber-rich foods such as fruits, whole grains, and vegetables, can also nourish beneficial gut bacteria [148]. By fostering the growth of advantageous bacteria and promoting SCFA production, the DASH diet may enhance gut health and potentially reduce the risk of CVD [149]. Additionally, vegan diets rich in fiber have been linked to increased SCFA production, supporting overall cardiometabolic health [150].

Several human studies, such as the GLYNDIET study [151], METADIET study [152], and METDIET study [153], have been established to explore the intricate interactions between diet, gut microbiome, and MetS. However, a notable gap exists in human studies designed to investigate the role of maternal diets in shaping the gut microbiota to prevent MetS in offspring.

Probiotic therapy involves the deliberate introduction of beneficial microbes into the gut microbiota [154]. Prebiotics, on the other hand, refer to food ingredients that promote the growth or enhance the activity of beneficial microorganisms [155]. Probiotics and prebiotics are commonly discussed and implemented in clinical practice. The term “synbiotic” is used when a product contains both probiotics and prebiotics. Additionally, postbiotics and parabiotics serve as beneficial agents promoting human health [156]. Postbiotics refer to the metabolites of probiotics after processing, such as vitamins, secreted proteins, SCFAs, and secreted biosurfactants. Parabiotics involve crude cell extracts or inanimate microbial cells of probiotics. An alternative method for re-establishing gut microbiota is fecal microbiota transplantation (FMT). This procedure entails the transfer of fecal material from a healthy donor to a recipient with the aim of restoring or altering the recipient’s gut microbiota. While certain research indicates the potential effectiveness of FMT in addressing obesity and its linked metabolic disorders [157], the existing evidence from both animal studies and rigorously controlled human trials is limited in confirming the benefits of FMT for improving metabolic parameters. Bacterial metabolite modulation is also a targeted therapy aiming to alleviate illness, for instance via the modulation of TMAO by microbial choline TMA lyase inhibitors, such as 3,3-dimethyl-1-butanol (DMB) or iodomethylcholine (IMC) [158,159].

As discussed in other scholarly works [160], evidence derived from diverse animal models of MetS programming reinforces the idea that therapies directed at the gut microbiota could potentially prevent MetS traits in adult offspring. Nevertheless, numerous aspects still lack clarity, particularly regarding the intricate interplay between the gut microbiota and oxidative stress in the context of MetS programming. Table 2 provides insight into studies that showcase gut-microbiota-targeted therapies in animal models, specifically those associated with oxidative stress (as presented in Table 1).

### 5.2. Targeting Gut Microbiota to Prevent Oxidative-Stress-Associated MetS Programming

Interventions targeting the gut microbiota have been explored in various models related to oxidative-stress-associated MetS programming. These models encompass scenarios such as protein restriction [161], maternal high-fructose diet [162,163], high-fat diet [72,117,122,164,165,166,167,168,169], maternal high-sucrose/fat diet [170], and maternal exposure to bisphenol A (BPA) [103,171,172].

**Figure 2:**
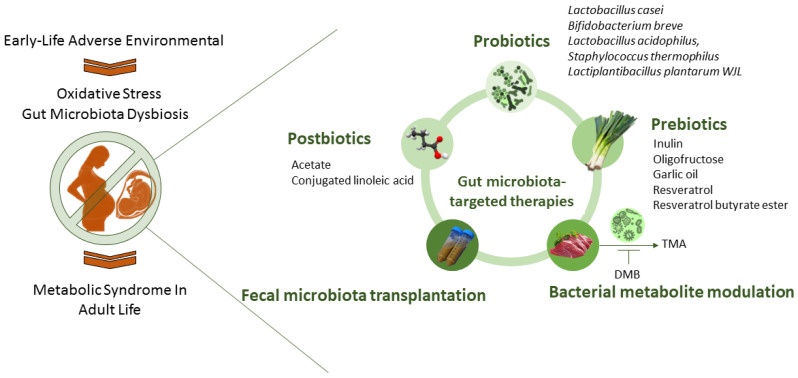
An overview of potential therapies targeting the gut microbiota to prevent the developmental programming of metabolic syndrome.

These gut-microbiota-targeted interventions involve the utilization of probiotics, prebiotics, and postbiotics, and the modulation of bacterial metabolites. A visual representation of gut-microbiota-targeted therapies for oxidative-stress-associated MetS programming is presented in Figure 2.

Fructose-primed developmental programming is intricately associated with both oxidative stress and the gut microbiota [173]. The consumption of high levels of fructose contributes to dysbiosis in gut microbial communities. These communities play a crucial role in maintaining host–microbiota homeostasis through redox signaling, and an imbalanced redox state can trigger inflammatory responses, leading to gut microbial dysbiosis [174]. Therapies such as probiotics, prebiotics, postbiotics, and the modulation of bacterial metabolites have shown promise in preventing fructose-induced MetS programming.

Studies have indicated the beneficial effects of probiotics in MetS [175]. For instance, supplementation with *Lactobacillus casei* during gestation and lactation prevented hypertension in adult offspring born to dams fed a high-fructose diet [162]. Additionally, the perinatal supplementation of inulin, a well-known prebiotic, provided protection against hypertension programmed by a maternal high-fructose diet in adult progeny [162]. Inulin’s beneficial action is associated with increased plasma propionate and the restoration of reduced GPR43 expression induced by a high-fructose diet.

Postbiotics, which include various constituents such as SCFAs, have also shown promise [176]. Acetate, an abundant SCFA, can interact with its receptors to regulate BP [177]. Perinatal acetate supplementation demonstrated benefits against offspring hypertension in a rodent model of a maternal high-fructose diet [163]. Furthermore, the modulation of the microbial metabolite TMAO has been effective in protecting against fructose-induced offspring hypertension. TMAO, transformed from trimethylamine (TMA), has been linked to CVD risk [178,179]. The inhibition of microbe-dependent TMAO and TMA formation [180], achieved by using the choline analog DMB, protected adult rat offspring against maternal high-fructose diet-programmed hypertension [163].

Gut-microbiota-targeted therapies have also been investigated in the context of a high-fat diet, another commonly used model for studying MetS programming. Probiotics, such as *Lactobacillus casei* and *Lactiplantibacillus plantarum* WJL, demonstrated reprogramming effects by improving gut microbiota diversity, hypertension, lipid profile, and insulin resistance in adult offspring [117,122,164]. Prebiotics, including inulin and oligofructose, have been effective in protecting against maternal high-fat diet-primed hypertension and improving hepatic steatosis, insulin sensitivity, and glucose tolerance in adult offspring [122,170].

Certain dietary components, including garlic and resveratrol, demonstrate prebiotic properties in addition to fibers [181,182]. While numerous foods are recognized as prebiotics, Table 2 highlights that only garlic and resveratrol have been studied in the context of MetS programming. Garlic, known scientifically as Allium sativum, is abundant in polysulfides, serving as a dietary source of hydrogen sulfide (H_2_S) donors [183] and contributing health benefits. Maternal supplementation with garlic oil has shown positive effects against hypertension in offspring primed with a high-fat diet. This supplementation led to increased levels of acetate, butyrate, and propionate in plasma, enhanced α-diversity, and an elevated proportion of beneficial microbes such as *Bifidobacterium* and *Lactobacillus* [72].

Resveratrol, a natural polyphenol found in grapes, is widely acknowledged for its antioxidant and prebiotic properties [184]. It has been proposed as a reprogramming strategy to prevent MetS traits [185,186]. Table 2 indicates that resveratrol has beneficial effects against MetS induced by a high-fat diet, addressing issues such as obesity, hyperlipidemia, and hepatic steatosis [165,166,168,169]. In a model combining maternal high-fat diet and NO deficiency [187], resveratrol protected adult offspring from hypertension by reducing the F/B ratio, a microbial marker associated with hypertension [188]. This highlights resveratrol’s potential to reshape the gut microbiome and prevent hypertension in offspring. Another example of a postbiotic used for reprogramming in MetS programming is conjugated linoleic acid. Derived from the gut microbiota metabolism, conjugated linoleic acid is catabolized from dietary polyunsaturated fatty acids. Maternal supplementation with conjugated linoleic acid has been shown to reverse cardiometabolic dysfunction in adult offspring primed by a high-fat diet [167].

Despite the benefits, the low bioavailability of resveratrol poses a challenge in translating basic scientific findings into clinical practice [189]. To address this concern, previous efforts have involved esterifying resveratrol with butyrate to create resveratrol butyrate esters (RBEs), aiming to enhance efficacy [190]. Research has demonstrated that low-dose RBEs (30 mg/L) improved hyperlipidemia and obesity in female progeny and hepatic steatosis in male offspring, both of which were primed by maternal exposure to bisphenol A (BPA) [171,172], showing sex-specific effects. Despite the known benefits of various prebiotic foods for MetS-related disorders [140], much remains unclear regarding their impact as reprogramming strategies for preventing offspring MetS.

Beyond resveratrol, recent research has highlighted the therapeutic potential of other dietary polyphenols in addressing obesity and related diseases [191,192]. Among these commonly consumed polyphenols are epigallocatechin [193], curcumin [194], quercetin [195], and epigallocatechin gallate [196]. Given that specific polyphenols have been identified as reprogramming agents to mitigate offspring hypertension [93], further investigation is warranted to elucidate whether they possess the capacity to prevent MetS programming.

### 5.3. The Interplay between Oxidative Stress and Gut Microbiota

In the preceding sections, we elucidated the pivotal roles of gut-microbiota-targeted therapy in mitigating oxidative-stress-associated MetS programming. Conversely, the utilization of antioxidant therapy in early life has exhibited promise for preventing MetS [46]. Various antioxidants administered during pregnancy and lactation in diverse models of MetS programming encompass vitamins, N-acetylcysteine (NAC), amino acids, polyphenols, melatonin, and synthetic antioxidants.

In a rodent model subjected to a high-fructose diet, perinatal supplementation with resveratrol prevented offspring hypertension while concurrently reducing oxidative stress and inducing alterations in gut microbiota composition [197]. Resveratrol therapy was found to augment the expression of nuclear factor erythroid-derived 2-related factor 2 (Nrf2) and decrease the expression of 8-OHdG in the offspring’s kidneys. Furthermore, the enduring effects of resveratrol on the offspring’s gut microbiota included an increase in the proportions of *Lactobacillus* and *Bifidobacterium* [197].

Another study demonstrated that antioxidant therapy with NAC in pregnant spontaneously hypertensive rats (SHRs) successfully prevented offspring hypertension [198]. The positive impact of maternal NAC therapy on hypertension was associated with a high abundance of the phylum *Actinobacteria* and the genera *Bifidobacterium* and *Allobaculum,* along with a low abundance of the phylum *Verrucomicrobia* and the genera *Akkermansia* and *Turicibacter*.

Additionally, L-malic acid, an antioxidant component commonly found in food additives, was investigated. A maternal diet supplemented with L-malic acid augmented antioxidant capacity, subsequently enhancing insulin sensitivity and glucose metabolism by modulating the gut microbiota of piglet offspring [199]. The increased abundance of *Romboutsia, Colidextribacter*, and *Family_XIII_AD3011_group* was associated with improved antioxidant capacity and glucose metabolism. Conversely, lower levels of *Blautia, Prevotellaceae_NK3B31_group, Prevotella,* and *Collinsella* were linked to reduced insulin sensitivity. This evidence underscores the intricate interplay between oxidative stress and the gut microbiota in the context of MetS programming.

### 5.4. Bridging the Gap between Animal Models and Clinical Practice

Animal research provides support for the potential preventive effects of the early utilization of specific gut-microbiota-targeted therapies against MetS programming. However, the translation of this growing body of evidence into clinical practice is still pending. Presently, the application of probiotics or prebiotics during gestation remains limited in human studies [200]. The evidence is scarce regarding probiotic supplementation for pregnant women, but the existing literature suggests its generally safe use and potential benefits for conditions such as gestational diabetes [201], preeclampsia [202], spontaneous preterm delivery [203], vaginal infections [204], and obesity [205]. However, information regarding the utility of prebiotic-rich foods or prebiotic-like components, either alone or in combination, during pregnancy is largely lacking [206].

It is crucial to note that there is a dearth of information on the influence of probiotic or prebiotic supplementation during gestation on the long-term outcomes of offspring related to MetS in human studies. Despite ongoing trials involving more than 10 studies focusing on prebiotic or probiotic supplements in pregnant women [207], none of them primarily concentrate on the development of MetS in offspring.

Elaborating on safety considerations, it is essential to emphasize that parabiotics and postbiotics are generally considered safer alternatives compared to probiotics. However, a notable gap exists in the realm of clinical practice, where a universally accepted definition for both parabiotics and postbiotics is still lacking. In contrast, authoritative bodies such as the Food and Agriculture Organization of the United Nations-WHO (FAO-WHO) and the International Scientific Association for Probiotics and Prebiotics (ISAPP) have established clear definitions for probiotics and prebiotics.

Given the dynamic nature of microbial-based therapies, there is a pressing need for governmental regulatory bodies to establish precise definitions for parabiotics and postbiotics from a regulatory standpoint. Clarity in terminology is crucial not only for effective communication within scientific and medical communities, but also for ensuring that regulatory frameworks can thoroughly assess and oversee the safety and efficacy of these emerging interventions. The absence of standardized definitions may introduce ambiguity, impeding the progress of research and application in these promising fields. Addressing this need for precise terminology will contribute to a more informed and regulated integration of parabiotics and postbiotics into clinical practice, promoting both scientific advancement and public health.

## 6. Concluding Remarks

Existing evidence indicates that the gut microbiota and oxidative stress play crucial roles as pathogenic factors in the developmental programming of MetS. Our review underscores the potential of gut-microbiota-targeted therapy as a strategy for reprogramming, aiming to prevent MetS. Despite the apparent benefits observed in MetS programming with this therapy, its effectiveness awaits confirmation through future human investigations. We anticipate that novel reprogramming interventions, influencing the interaction between oxidative stress and gut microbiota, will emerge in the future, contributing to the prevention of MetS.

## Figures and Tables

**Table 1:** Offspring MetS in animal models related to oxidative stress.

Strain	Sex	Animal Models	Protocol	Age at Measure (Weeks)	Outcome	Mechanisms of Oxidative Stress
SD/Wistar	M	Caloric restriction	50% of ad lib	12–16	↑ BP [61,62]; insulin resistance [63]	↑ Renal 8-OHdG expression, ↑ ADMA, ↓ NO, [61]; ↓ NO, ↑ 3-NT [62]
Wistar	M	Protein restriction	8–9% protein	12	↑ BP [64]; insulin resistance [65]	↑ F_2_-isoprostane, ↓ glutathione [64]
SD/C57BL/6J	M + F	Maternal high-fructose diet	60% fructose [66,67]; 10% fructose solution [68]	12–52	↑ BP, insulin resistance, and dyslipidemia [66,67,68]; ↑ adiposity [68]	↓ NO, ↑ renal 8-OHdG expression [69]; ↑ MDA, ↑ brain NADPH-oxidase expression [70]; ↑ ROS [71]
SD/Wistar	M + F	Maternal high-fat diet	58% fat [72,73,74,75,76]; 31% fat/cholesterol [77]	14–16	↑ BP [72]; ↑ adiposity [73,75]; dyslipidemia [74,75] and hyperinsulinemia [75]	↓ SOD activity in M; ↑ renal MDA level in F [76]; ↑ renal 8-OHdG expression [77]
SD/Wistar	M	Maternal diabetes	STZ (45 mg/kg, i.p.) at day 0 of gestation [78]; STZ (50 mg/kg, i.p.) at day 1 after birth [79]; STZ (120 mg/kg, i.p.) on postnatal day 5 [79,80]	12–16	↑ BP [78]; ↑ adiposity [79]; insulin resistance and dyslipidemia [80]	↑ ADMA, ↓ NO [78]; ↑ renal 3-NT and TBARS [81]; ↑ ROS, ↓ SOD activity, ↓ NO [82]
SD/Wistar	M + F	Maternal inflammation	LPS (0.79 mg/kg, i.p.) at days 8, 10 and 12 of gestation [83]; surgically induced periodontitis 13 days before mating [84]	11–12	↑ BP [83]; insulin resistance [84]	↑ Renal MDA [85]
WKY/Wistar	M	Uteroplacental insufficiency	Bilateral uterine artery ligation on gestational day 18 [86] or 19 [87]	22–30	↑ BP [86]; dyslipidemia and insulin resistance [87]	↑ Urinary F_2_-isoprostane level, ↑ renal NADPH-oxidase-dependent superoxide [88]
SD	M	Maternal stress	DEX (0.2 mg/kg, i.p.) at days 15 and 16 of gestation [89]; DEX (0.1 mg/kg, i.p.) at gestational days 14 to 20 [90,91]	16–24	↑ BP [89,90]; ↑ adiposity and insulin resistance [90]	↓ Renal NO [89]; ↓ Gpx1 expression, ↑ NADPH-oxidase [92]; ↑ ADMA, ↑ renal 8-OHdG expression, [93]
SD/Wistar	M	Maternal chronodisruption	Continuous light exposure [94]; continuous light exposure at days 12 to 21 of gestation [95]	12–18	↑ BP [100], insulin resistance [95]	↑ Brain ROS [96]
SD/Wistar	M + F	Maternal nicotine exposure	Nicotine (4 µg/kg/min) from gestational day 4 to postnatal day 10 [97,98]; nicotine (6 mg/kg/day) at postnatal days 2 to 16 [99]	20–32	↑ BP [97,98]; hyperlipidemia and steatosis [99]	↑ MDA, 3-NT, and NADPH oxidase [97]; ↑ 4-NHE and MDA levels, ↓ GPx1 activity [99]
SD	M + F	Maternal ethanol exposure	Administration of 1 g of ethanol/kg through oral gavage on gestational day 13 and 14 [100,101]	24	↑ BP [100]; insulin resistance [101]	↓ SOD1, CAT, and Gpx1; ↑ NOX2 [102]
SD	M + F	Prenatal BPA exposure	Oral gavage with 50 μg/kg BPA [103]; oral 240 μg/kg BPA [104]	16–24	↑ BP [103]; insulin resistance [104]	↑ Renal 8-OHdG expression, ↑ ADMA, ↓ NO [103]
SD/Wistar	M	Maternal DEHP exposure	Administration of 6.25 mg/kg DEHP through oral gavage [105]; oral gavage with 100 mg/kg DEHP from gestational day 9 to postnatal day 21 [106]	12–21	↑ BP [105]; insulin resistance [106]	↑ Renal ROS [107]

SD = Sprague Dawley rat; WKY = Wistar Kyoto; M = male; F = female; STZ = streptozotocin; LPS = lipopolysaccharide; DEX = dexamethasone; i.p. = intraperitoneal injection; BPA = bisphenol A; DEHP = di-n-butyl phthalate; ADMA = asymmetric dimethylarginine; NO = nitric oxide; 8-OhdG = 8-hydroxy-2′–deoxyguanosine; CAT = catalase; ROS = reactive oxygen species; 3-NT = 3-nitrotyrosine; TBARS = thiobarbituric acid; 4-NHE = 4-hydroxynonenal; Gpx1 = glutathione peroxidase 1; SOD= superoxidase dismutase; MDA = malondialdehyde; NOX2 = NADPH oxidase 2.

**Table 2:** Summary of animal models illustrating therapies targeting the gut microbiota for metabolic syndrome with developmental origins.

Animal Models	Gut-Microbiota-Targeted Therapies	Strain/Sex	Age at Measure (Weeks)	Outcomes	Ref.
Protein restriction	Resveratrol (20 mg/kg/day) during pregnancy	Wistar/M + F	16	Improved insulin resistance and obesity	[161]
Maternal high-fructose diet	*Lactobacillus casei* via oral gavage during pregnancy and lactation	SD/M	12	↓ BP	[162]
Maternal high-fructose diet	5% *w*/*w* long-chain inulin during pregnancy and lactation	SD/M	12	↓ BP	[162]
Maternal high-fructose diet	Administration of magnesium acetate (200 mmol/L) through drinking water throughout the pregnancy and lactation periods	SD rat/M	12	↓ BP	[163]
Maternal high-fructose diet	Administration of 1% DMB through drinking water throughout the pregnancy and lactation periods	SD rat/M	12	↓ BP	[163]
Maternal high-fat diet	Multi-strain probiotics via oral gavage during pregnancy and lactation	C57BL/6 J/F	20	Improved glucose and insulin levels	[164]
Maternal high-fat diet	Administration of resveratrol (50 mg/L) through drinking water throughout the pregnancy and lactation periods	Wistar/M + F	3	Improved obesity	[165]
Maternal high-fat diet	Resveratrol (0.2% *w*/*w*) during pregnancy and lactation	C57BL/6 J/M	14	Improved obesity and hyperlipidemia	[166]
Maternal high-fat diet	1% conjugated linoleic acid in chow during pregnancy and lactation	SD/M	21	Improved cardiometabolic dysfunction	[167]
Maternal high-fat/high-cholesterol diet	*Lactiplantibacillus plantarum WJL* via oral gavage during pregnancy and lactation	Wistar/M	13	↓ BP, improved insulin resistance and hyperlipidemia	[117]
Maternal and post-weaning high-fat diet	*Lactobacillus casei* via oral gavage during pregnancy and lactation	SD/M	16	↓ BP	[122]
Maternal and post-weaning high-fat diet	5% *w*/*w* long-chain inulin during pregnancy and lactation	SD/M	16	↓ BP	[122]
Maternal and post-weaning high-fat diet	Garlic oil (100 mg/kg/day) via oral gavage during pregnancy and lactation	SD/M	16	↓ BP	[72]
Maternal and post-weaning high-fat diet	Administration of resveratrol (50 mg/L) through drinking water throughout the pregnancy and lactation periods	SD/M	16	↓ BP	[168]
Maternal and post-weaning high-fat diet	Administration of resveratrol (50 mg/L) through drinking water throughout the pregnancy and lactation periods	SD/M	16	Improved hyperlipidemia, obesity, and hepatic steatosis	[169]
Maternal high-fat/sucrose diet	10% *w*/*w* oligofructose during pregnancy and lactation	SD/M	24	Improved insulin sensitivity, glucose tolerance, and hepatic steatosis	[170]
Maternal BPA exposure and high-fat diet	Administration of resveratrol (50 mg/L) through drinking water throughout the pregnancy and lactation periods	SD/M	16	↓ BP	[103]
Maternal BPA exposure	Resveratrol butyrate ester (30 or 50 mg/kg/day) via oral gavage during pregnancy and lactation	SD/F	7	Improved obesity and hyperlipidemia	[171]
Maternal BPA exposure	Resveratrol butyrate ester (30 mg/kg/day) via oral gavage during pregnancy and lactation	SD/M	7	Improved hepatic steatosis	[172]

Studies tabulated based on animal models, type of intervention, and age at measure. BPA = bisphenol A; SD = Sprague Dawley rat; DMB = 3,3-maternal dimethyl-1-butanol.
